# Diffuse Large B-Cell Lymphoma with Calf Muscle Localization

**DOI:** 10.1155/2011/292494

**Published:** 2011-07-09

**Authors:** Laura Bourdeanu, Rashmi Menon, George Somlo

**Affiliations:** Department of Medical Oncology and Experimental Therapeutics, City of Hope Comprehensive Cancer Center, 1500 E Duarte Road, Duarte, CA 91010, USA

## Abstract

Although diffuse large B-cell lymphoma (DLBCL) usually occurs in the lymph nodes, approximately 30–40% of the time it can have an extranodal site of involvement and it can arise in nearly every body site such as intestine, bone, breast, liver, skin, lung, and central nervous system. Muscle involvement of DLBCL is especially uncommon, comprising 0.5% of extranodal NHL. We report a case of a 72-year-old man with extranodal DLBCL of a unique manifestation in the calf muscle, involving predominantly the gastrocnemius muscle. The patient achieved complete response and remained free of local recurrence or metastasis following diagnosis.

## 1. Introduction

Non-Hodgkins lymphoma (NHL) is the most common form of lymphoma, with an estimated 65,980 new cases of NHL diagnosed in America during 2009, resulting in approximately 19,500 NHL deaths [1]. Diffuse large B-cell lymphoma (DLBCL) is the most common form of NHL, accounting for more than one-third of all lymphomas [2]. Although DLBCL usually occurs in the lymph nodes, it can arise in other tissues such as intestine, bone, breast, liver, skin, lung, and the central nervous system. Muscle involvement of primary disease is especially uncommon, comprising 0.5% of extranodal NHL [3]. Although extranodal DLBCL can involve virtually any muscular structure, calf localization has not yet been reported. Here, we present a case report of a unique manifestation of DLBCL in the calf muscle, involving predominantly the gastrocnemius muscle.

## 2. Case Report

**Figure 1:**
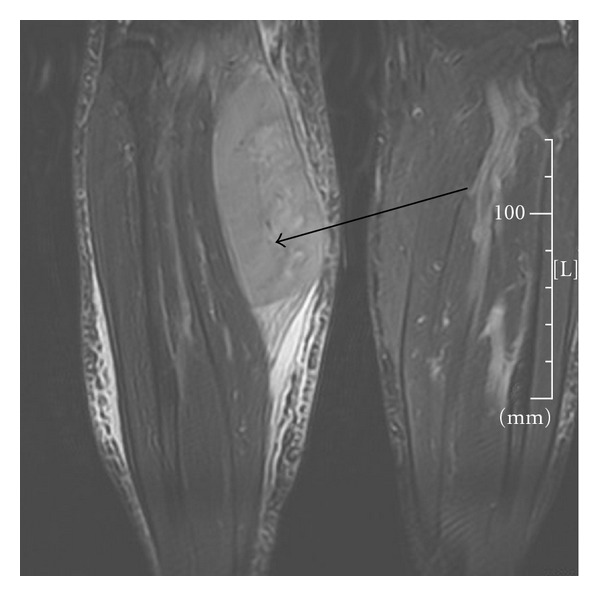
MRI of the lower extremities with right leg mass involving the medial gastrocnemius muscle.

A 72-year-old male initially presented to his general practitioner with complaints of right calf pain and swelling that started while doing maintenance on his roof. Physical examination revealed a firm mass in the right calf, measuring approximately 10 cm, nontender, with no warmth or erythema. Distally, the calf was grossly neurovascularly intact, with no inguinal adenopathy. Magnetic resonance imaging (MRI) of the right calf showed diffuse increased signal intensity of the medial gastrocnemius muscle that was associated with a central 3 cm region of abnormal signal intensity, possibly due to contusion and muscular injury. A repeat MRI in one or two months was advised to ensure stability of the findings. The repeat MRI showed marked enlargement, to 11.5 × 6.0 × 8.5 cm, of the mass involving the medial gastrocnemius muscle, suspicious for sarcoma (Figure 1). The adjacent osseous structures appeared intact and demonstrated no evidence of destructive changes to the bone. A whole-body FDG-PET scan revealed intense activity that involved the right gastrocnemius muscle, with no abnormal activity in the chest, abdomen, or the remainder of the lower extremities.

An incisional biopsy was obtained from the mass and stained with hematoxylin and eosin and immunostains specific to certain cancers. The biopsy tissue contained diffuse infiltrate of large malignant cells with a high nucleus-to-cytoplasm ratio and scanty cytoplasm. In addition, the nuclei were round, with prominent nucleoli and high mitotic activity. The differential diagnosis based on morphology included sarcoma, Merkel cell carcinoma, and lymphoma. Immunoperoxidase staining results were consistent with malignant B-cell lymphoma (Table 1). Laboratory measurements were all within normal limits, except for elevated values of lactate dehydrogenase 596 U/L (normal: 297–537), which is indicative of more advanced disease. Serum levels of soluble IL-2 receptors were not measured. 

Bone marrow aspiration revealed normocellular marrow (50%) with adequate trilineage hematopoesis, and no evidence of lymphoma, immunoglobulin heavy chain gene rearrangements, or immunoglobulin kappa light chain gene rearrangements. Bilateral marrow trephine biopsy sections were adequate and normocellular (50%) for age. Granulopoietic and erythroid maturation were adequately present with an M : E ratio of 3 : 1. Megakaryocytes were normal in number and appearance. No focal lesions were identified. Trabecular bone is normal. Cytogenetic analysis showed a male karyotype with loss of the Y chromosome in 4 of 20 mitotic cells, most likely an age-related phenomenon. No additional clonal cytogenetic changes were detected. Additionally, the 14q32/IGH translocation was not detected above established background limits by FISH analysis. 

The patient was started on aggressive chemotherapy with rituximab (375 mg/m^2^), doxorubicin (50 mg/m^2^), vincristine (1.4 mg/m^2^), and cyclophosphamide (750 mg/m^2^). The chemotherapy was administered intravenously every three weeks for a total of six cycles. Subsequently, the patient received three cycles of rituximab (375 mg/m^2^) administered intravenously every three months. In addition, the patient received radiation therapy via 6-field IMRT technique. A dose of 32.4 Gy was given in eighteen 1.8 Gy fractions. The radiotherapy field was then reduced to a smaller field which covered the MRI-defined postchemotherapy residual tumor, and an additional 12.6 Gy was given to this volume. This brought the total radiation dose of the residual disease to 45 Gy. Six MV photons were used for all treatments. 

The follow-up PET scan obtained one month after the end of treatment showed tremendously decreased activity in the right lower extremity, with only a residual amount of activity noted along the medial gastrocnemius muscle that could have been caused by some mild physiological activity. The posttreatment serum LDH value was 407 U/L. The subsequent CT scan reported some residual hypoattenuation involving the proximal medial gastrocnemius muscle and loss of muscle mass consistent with muscle atrophy. Otherwise, there was no evidence of soft tissue masses or fluid collection. The one-year follow-up PET scan revealed no evidence of disease recurrence.

## 3. Discussion

DLBCL is an aggressive form of non-Hodgkins lymphoma, and comprises approximately 30% of all lymphomas. This type of lymphoma occurs predominantly in men over 64 years of age and typically presents as a fast-growing lymph node or mass with or without “B” symptoms, including fever, weight loss, and night sweats. Extranodal presentation most commonly involves the gastrointestinal tract or bone marrow, and less commonly occurs in the skeletal muscle. When skeletal muscle involvement is seen, it is predominantly in upper arm muscles and glutei [4]. The relative 5-year survival rate in patients with extranodal presentation is 50% [2]. The standard treatment for DLBCL is combination chemotherapy and immunotherapy, known as R-CHOP (rituximab, doxorubicin, cyclophosphamide, vincristine, and prednisone) in combination with radiation therapy [5]. 

This patient presented with a fast-growing right calf mass, without “B” symptoms. Upon biopsy, the patient was found to have stage IA_ E _ DLBCL, in which the lymphoma was limited to one site and had not spread to other organs or lymph nodes. The biologic grade was high grade, with a proliferation index of 70% as measured by Ki67 expression. The patient received 6 cycles of R-CHOP, without any treatment-related complications, followed by radiation and three subsequent rituximab infusions. He achieved complete response and remained free of local recurrence or metastasis in the 16 months following diagnosis. This supports the concept that correct diagnosis and early chemotherapy with R-CHOP and radiation, followed by rituximab infusions, are appropriate treatments in early-stage primary skeletal muscle lymphoma.

## Figures and Tables

**Table 1:** Immunoperoxidase stains.

Antibody	Result
Pancytokeratin	Negative
CD3	Scattered cells stained
CD20	Strongly positive
CD79a	Weakly positive
Synaptophysin	Negative
Ki67	70% of cells stained
CD99	Negative
S100 protein	Negative
Epithelial membrane antigen	Negative
